# Intra- and inter-session reliability of electrical detection and pain thresholds of cutaneous and muscle primary afferents in the lower back of healthy individuals

**DOI:** 10.1007/s00424-023-02851-7

**Published:** 2023-08-25

**Authors:** Daniel Streuli, Luana Nyirö, Jan Rosner, Andreas Schilder, Miklos Csato, Petra Schweinhardt

**Affiliations:** 1 Department of Chiropractic Medicine, Integrative Spinal Research, Balgrist University Hospital, University of Zurich, Zurich, Switzerland; 2 Spinal Cord Injury Center, Balgrist University Hospital, University of Zurich, Zurich, Switzerland; 3 Department of Neurology, University Hospital Bern, Inselspital, University of Bern, Bern, Switzerland; 4 Department of Orthopaedics and Trauma Surgery, Medical Faculty Mannheim, Heidelberg University, Mannheim, Germany; 5 Department of Radiology, Balgrist University Hospital, University of Zurich, Zurich, Switzerland

**Keywords:** Low back pain, Experimental pain models, Electrical stimulation, Deep tissue afferents, Muscle pain, Quantitative sensory testing

## Abstract

To advance evidence-based practice and targeted treatments of low back pain (LBP), a better pathophysiological understanding and reliable outcome measures are required. The processing of nociceptive information from deeper somatic structures (e.g., muscle, fascia) might play an essential role in the pathophysiology of LBP. In this study, we measured the intra- and inter-session reliability of electrical detection and pain thresholds of cutaneous and muscle primary afferents of the lower back. Twenty healthy participants attended two study visits separated by 27.7 ± 1.7 days. To determine the location-specific electrical detection threshold (EDT) and pain threshold (EPT), needle electrodes were inserted in the epidermal layer over, and in the lumbar erector spinae muscle. Additionally, established quantitative sensory testing (QST) parameters were assessed. Reliability was determined by differences between measurements, intraclass correlation coefficients (ICC_2,1_), Bland–Altman plots, and standard error of measurement (SEM). Correspondence between QST parameters and electrical thresholds was assessed using Pearson’s correlation. Except for cutaneous EPT, no significant (*p* ≤ 0.05) intra- and inter-session differences were observed. Excellent intra-session reliability was shown for cutaneous and intramuscular electrical stimulations and all QST parameters (ICC: 0.76–0.93). Inter-session reliabilities were good (ICC: 0.74–0.75) except for electrical stimulations (ICC: 0.08–0.36). Limits of agreement and SEM were higher for inter-session than intra-session. A medium to strong relationship was found between electrical and mechanical/pressure pain thresholds. In conclusion, cutaneous and intramuscular electrical stimulation will potentially close an important diagnostic gap regarding the selective examination of deep tissue afferents and provide location-specific information for the excitability of non-nociceptive and nociceptive afferents.

**Supplementary Information:**

The online version contains supplementary material available at 10.1007/s00424-023-02851-7.

## Introduction

Most low back pain (LBP) patients suffer from non-specific LBP, i.e., pain without a discernable pathoanatomical source [22]. Still, in most cases of non-specific LBP, nociceptive fibers in deep spinal soft tissue (e.g., muscle, fascia) contribute to the generation of pain, in addition to important sources such as intervertebral discs and facet joints, which are the main targets for LBP treatments [6, 13, 38, 39, 45]. It is supposed that localized nociceptive input can develop into widespread pain via central sensitization (CS) processes at spinal and supraspinal levels [32]. In support of spinal CS, segmental hypersensitivity, as assessed using temporal summation of pain (TSP), has been demonstrated in patients with chronic pain conditions such as neuropathic, musculoskeletal and visceral pain [4]. Further, descending facilitation of pain from the rostral ventromedial medulla has been observed for neuropathic pain in rats after spinal nerve ligation [44], and impaired descending pain control, arising supraspinally and assessed in humans via conditioned pain modulation (CPM) protocols, contributes to central sensitization [19, 32]. Quantitative sensory testing (QST) allows the assessment of modality-specific primary somatosensory afferent fibers, thereby aiding patient characterization [15, 16, 36, 37]. However, the investigation of primary afferent fibers from deep soft tissues is scarce as QST protocols are biased towards assessing skin afferents [30]. This is unfortunate, given that there are indications that nociceptive information from skin and deep tissue are processed differently: TSP [27] as well as descending pain modulation effects [46, 47] have been found to be pronounced in response to stimulation of deep tissue afferents compared to superficial afferents in the skin. In order to examine potentially different processing from deep soft tissues and its relevance for LBP, it is necessary to selectively stimulate deep primary afferents and to contrast this with cutaneous stimulation. Electrical stimulation of muscle or fascia might allow to more selectively stimulate deep soft tissue, without affecting cutaneous layers [39]. This method has been employed in a few studies [1, 21, 39], and some observations on inter-session reliability have been reported in the groin [1], but not in the lower back or in comparison to other QST parameters. A pre-requisite for the comparison of cutaneous vs. intra-muscular electrical stimulation in, e.g., a CPM paradigm would be a comprehensive assessment of the intra- and inter-session reliability. To our knowledge, no test–retest reliability study of electrical detection thresholds (EDT) and electrical pain thresholds (EPT) within cutaneous and muscle tissue at the lower back has been conducted.

Therefore, the current study assesses the intra- and inter-session reliability of cutaneous and muscle primary afferents from the lower back of healthy pain-free participants. This is compared to the reliability of well-established QST paradigms, i.e., mechanical detection (MDT), mechanical pain thresholds (MPT), and pressure pain thresholds (PPT), acquired in the same participants.

## Methods

### Participants

Participants were recruited between May 2021 and April 2022 through the online platform “https://marktplatz.uzhalumni.ch” and an advertisement in the University of Zurich student newspaper. Inclusion criteria were being between 18 and 40 years of age, proficient in German or English to understand the instructions, and signing the informed consent. Exclusion criteria were acute pain; more than 3 consecutive days of back pain in the last year; regular or current intake of pain medication; any neurological, major medical, psychiatric, or chronic pain condition; or pregnancy. The Independent Ethics Committee of the Canton of Zurich approved the study protocol (Ethics-Nr: 2019–00473). All participants gave written informed consent prior to study participation.

### Study design

**Fig. 1:**
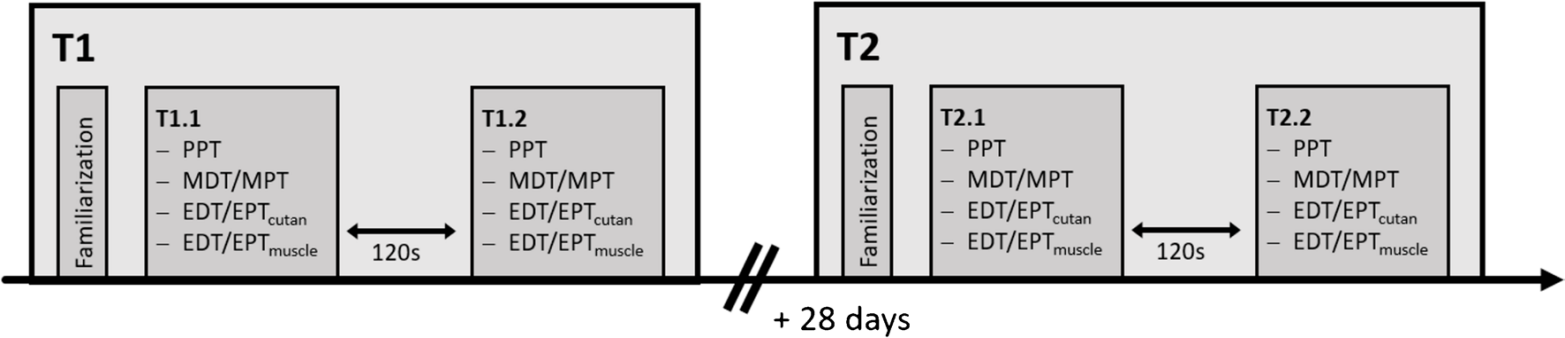
Experimental protocol. Measurement sessions T1 and T2 separated by 28 days. EDT, electrical detection threshold; EPT, electrical pain threshold (muscle, intramuscular; cutan, epidermis/dermis); MDT, mechanical detection threshold; MPT, mechanical pain threshold; PPT, pressure pain threshold

**Fig. 2:**
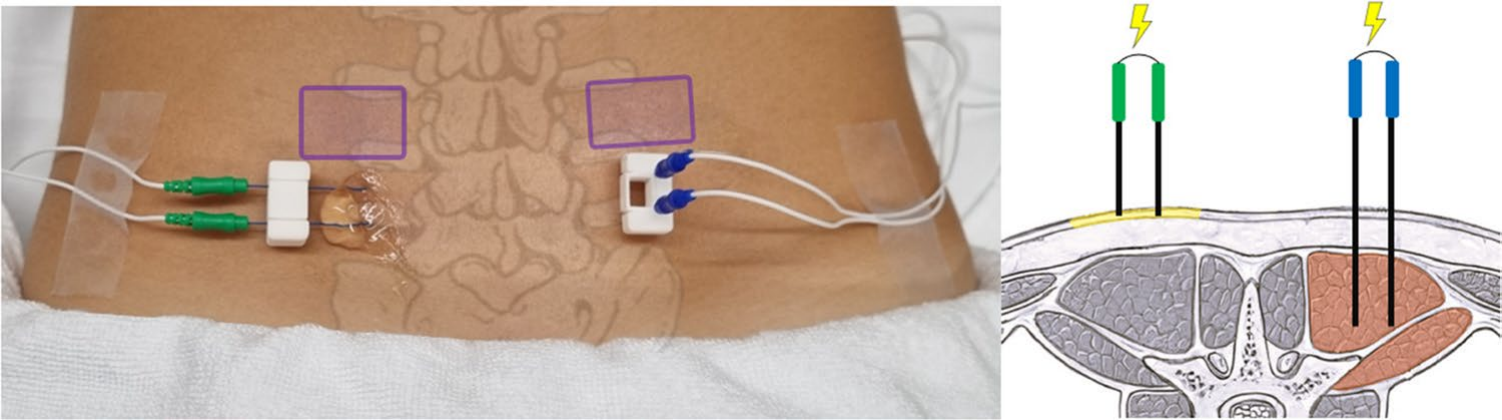
Location and positioning of the concentric needle electrodes. Green: cutaneous electrodes; blue: intramuscular electrodes; purple: area for determination of mechanical and pressure thresholds

A clinimetric study examining the test–retest reliability of EDT and EPT of cutaneous and muscle tissue from the lower back was conducted. Each participant attended in two sessions (T1 and T2) separated by 28 days (+ / − 5 days) to decrease the inter-session variability due to the menstrual cycle for women [34, 42]. The time of day was kept constant across the two sessions to eliminate variability related to circadian rhythm [11]. In the two sessions, EDT and EPT measurements in cutaneous (i.e. epidermis/dermis) and deep (i.e. muscle/fascia) tissue of the lower back were performed. In addition, MDT, MPT, and PPT measurements were conducted in the lower back, following the QST protocol of the German Research Network of Neuropathic Pain (DFNS) [37]. Every stimulation modality (EDT/EPT_muscle_, EDT/EPT_cutan_, MDT/MPT, and PPT) was measured in a designated area, at the facet joint level of L3/4 resp. L4/5 on longitudinal axis and the maximal sagittal thickness of the erector spinae muscle on the transverse axis (see Fig. 2). The exact location was determined with ultrasound and marked using a skin marker. The location and order of the four different modalities were counter-balanced and randomized but were kept the same for T1 and T2 of a given participant. After measuring all modalities in session T1 resp. T2 (first measurement on day 1: T1.1; first measurement on day 2: T2.1), there was a 120-s break (see Fig. 1). Thereafter, all modalities were measured again in the same order and location (second measurement on day 1: T1.2; second measurement on day 2: T2.2). The non-electrical stimulations MDT, MPT, and PPT are subsumed under the term “QST parameters.”

The measurements were conducted in a quiet room with a controlled room temperature (24.1 ±1.4 °C). All measurements were performed by the same pair of investigators in the same roles.

For detection thresholds (MDT, EDT), the stimulus intensity when the participant just felt a sensation at the stimulation site was used [37, 39]. Individual pain thresholds (MPT, PPT, and EPT) were defined as the stimulus intensity at which an additional sensation such as pulling, burning, or pricking was felt [35].

At the beginning of every session, the participants were familiarized with the different modalities: MDT, MPT, and PPT were demonstrated at the hand and lumbar spine; EDT and EPT familiarization was performed with the participant in a prone position after positioning of the needles in the lower back as described in the following section.

### Cutaneous and intramuscular electrical detection and pain thresholds

Before positioning the needles, landmarks in the lower back were palpated (e.g., spinous process of L4 and L5), marked with a skin marker, and thereafter, the level of the vertebrae was verified with ultrasound.

For EDT/EPT_cutan_ and EDT/EPT_muscle_, two unipolar concentric needle electrodes (Neurolite AG, Belp, Switzerland) with 0.34 mm^2^ stimulation area were inserted bilaterally with a distance of 10 mm between the needle tips, ensured by a custom-made 3D-printed needle-guiding device (Online Resource Fig. S1), into the epidermis/dermis (see Fig. 2). With similar dimension of the needle electrode diameter and the thickness of the epidermis and furthermore the manual insertion of the needle by hand, there is a low probability of positioning the needle tip only within the epidermis. Therefore, electrical cutaneous stimulation in the present study is meant to be epidermal/dermal stimulation. The needle positioning for cutaneous stimulation within the epidermis/dermis was verified with ultrasound after insertion, and the dimension of the erector spinae muscle and the depth of the deep layer of the thoracolumbar fascia were measured with ultrasound before inserting the intramuscular needle electrodes.

The measurements for cutaneous and intramuscular electrical stimulations were performed using a Dantec Keypoint Focus System (Natus Neurology Incorporated, Wisconsin, USA). For threshold determination, a staircase approach was developed in pilot measurements to reduce the number of stimulations to minimize habituation effects.

Every staircase consisted of a series of single rectangular electrical impulses of 0.04 ms duration with an inter-stimulus interval of 3–5 s to avoid temporal summation [3, 4].

For the determination of cutaneous and intramuscular EDT, the staircase approach started at 0.5 mA and then increased by 0.2 mA steps until the detection threshold was reached or reduced until the sensation was lost.

The intensity for the cutaneous and intramuscular EPT commenced at 5.0 mA and was increased by 2.0 mA steps until the first perception of pain, using the definition described above. Afterwards, the steps were reduced to steps of 0.5 mA until the pain sensation was lost and increased until detected again. The participants were asked to describe the quality of cutaneous and intramuscular EPT after the applications to validate that the participants understood the concept of an additional sensation to determine the pain threshold. The maximum current to determine cutaneous and intramuscular EPT was limited to 45.0 mA for safety reasons to avoid potential tissue damage.

The final thresholds were the geometric mean of five up-and-down staircase stimulus intensities, equivalent to the guidelines for MDT and MPT of the DFNS [37] and as applied also in the present study.

### Mechanical detection threshold (MDT)

A standardized set of modified von Frey hairs (OptiHair_2_-Set, Marstock Nervtest, Germany), with forces from 0.25 to 512 mN in a factor two progression was used to measure MDT one vertebral level above the test sites for electrical stimulation, at the facet joint level L3/4 (see Fig. 2). The contact area of these von Frey hairs is of uniform size and shape (rounded tip, 0.5 mm in diameter). The filaments were applied in descending order starting at 16 mN until the sensation was lost and then increased until detected again.

### Mechanical pain threshold (MPT)

A set of calibrated pinprick devices (cylindrical tip, 0.25 mm diameter) with forces of 8–512 mN in a factor of two progression (MRC Systems GmbH, Heidelberg, Germany) was used to measure MPT in the same area as MDT. The pinprick devices were applied in ascending order until the first perception of pain/sharpness was detected, starting at 8mN. As for the EPTs, the participants were asked to describe the stimulus quality of the MPT after the applications.

### Pressure pain threshold (PPT)

The PPT was measured using a pressure algometer (manual: FDN 200®, Wagner Instruments, USA) laterally over the facet joint of L3/4 in a perpendicular projection of the maximal sagittal thickness of the erector spinae muscle (see Fig. 2) with a probe area of 1 cm^2^ following the guidelines of the DFNS [37]. The final PPT was calculated as the arithmetic mean of three series of slowly increasing stimulus intensities (+ 0.5 kg/s resp. 50 kPa/s) following the guidelines of the DFNS [25, 31]. In addition, the participants described the pain quality after the three applications.

### Data analysis

All modalities from measurement T1.1 were analyzed for distribution properties to determine whether data transformation was required to approximate normal distribution. For each stimulus modality, skewness, kurtosis, and Kolmogorov–Smirnov d statistics were determined for raw and log-transformed data. The geometric mean of skewness and kurtosis was multiplied by the Kolmogorov–Smirnov d as an attribute of normal distribution [14, 36]. In case the ratio for raw data to log-transformed data for any specific modality was equal to or greater than 3, then log-transformation was considered a better representation of normal distribution and was used for further analysis (Online Resource Table S1).

All statistical calculations were conducted with the open-source statistical computing language R with the software package RStudio version 2021.09.2 (Boston, MA, 2022) and several of its packages.

To determine intra- and inter-session test–retest reliability, differences between measurements, intraclass correlation coefficients, Bland–Altman plots, and standard error of measurement were investigated [17].

- Measurement differences between the first measurement of T1 (T1.1) and the second measurement of T1 (T1.2) and between the first measurement of T1 (T1.1) and the first measurement of T2 (T2.1) were analyzed with paired samples *t*-tests to determine whether the intra- and inter-session measurement differences are statistically different from zero.
- Intraclass correlation coefficients (ICCs) between T1.1–T1.2 and between T1.1–T2.1 were calculated to reflect the degree of correlation between measurements. The agreement was determined using a two-way random, absolute agreement (ICC 2.1). The ICC coefficients were graded as follows: ICC < 0.40 poor reliability, 0.40–0.59 fair reliability, 0.60–0.75 good reliability, and > 0.75 excellent reliability [41].
- Bland–Altman limits of agreement (LoA) plots between T1.1–T1.2 and between T1.1–T2.1 were used to evaluate and illustrate the level of agreement between intra- and inter-session measurements.
- The standard error of measurement (SEM) between T1.1–T1.2 and between T1.1–T2.1 was determined.

To determine the strength and direction of the correlation between electrical detection and pain thresholds and established QST measures, Pearson’s correlation coefficient was used. We considered 0.10 < *r* < 0.39 as small, 0.30 < *r* < 0.5 as medium, and 0.50 < *r* < 1.0 as strong correlations [12].

## Results

Twenty participants, 10 males (28.1 ± 4.7 years, mean ± SD) and 10 females (26.5 ± 1.9 years, mean ± SD), were enrolled in the study (Table 1).

**Table 1:** Descriptive characteristics of participants

Characteristics	Healthy participants		
	Total	Male	Female
Number of participants, *N*	20	10	10
Age, years, mean ± SD (range)	27.3 ± 3.6	28.1 ± 4.7	26.5 ± 1.9
	(22–39)	(22–39)	(23–29)

*SD*, standard deviation

All twenty participants completed the two sessions, which were separated by 27.7 ± 1.7 days (mean ± SD). For one participant, after applying the pressure gauge device for the PPT measurement in visit T1.1, the cutaneous needles got displaced and had to be repositioned for measurement T1.2. Therefore, the intra-session reliability of EDT/EPT_cutan_ consists of 19 participants. In another participant, the intramuscular needles were dislocated by a movement of the patient after the measurement T1.1; hence, the intra-session reliability of EDT/EPT_muscle_ consists also of 19 participants.

### Detection and pain thresholds

**Fig. 3:**
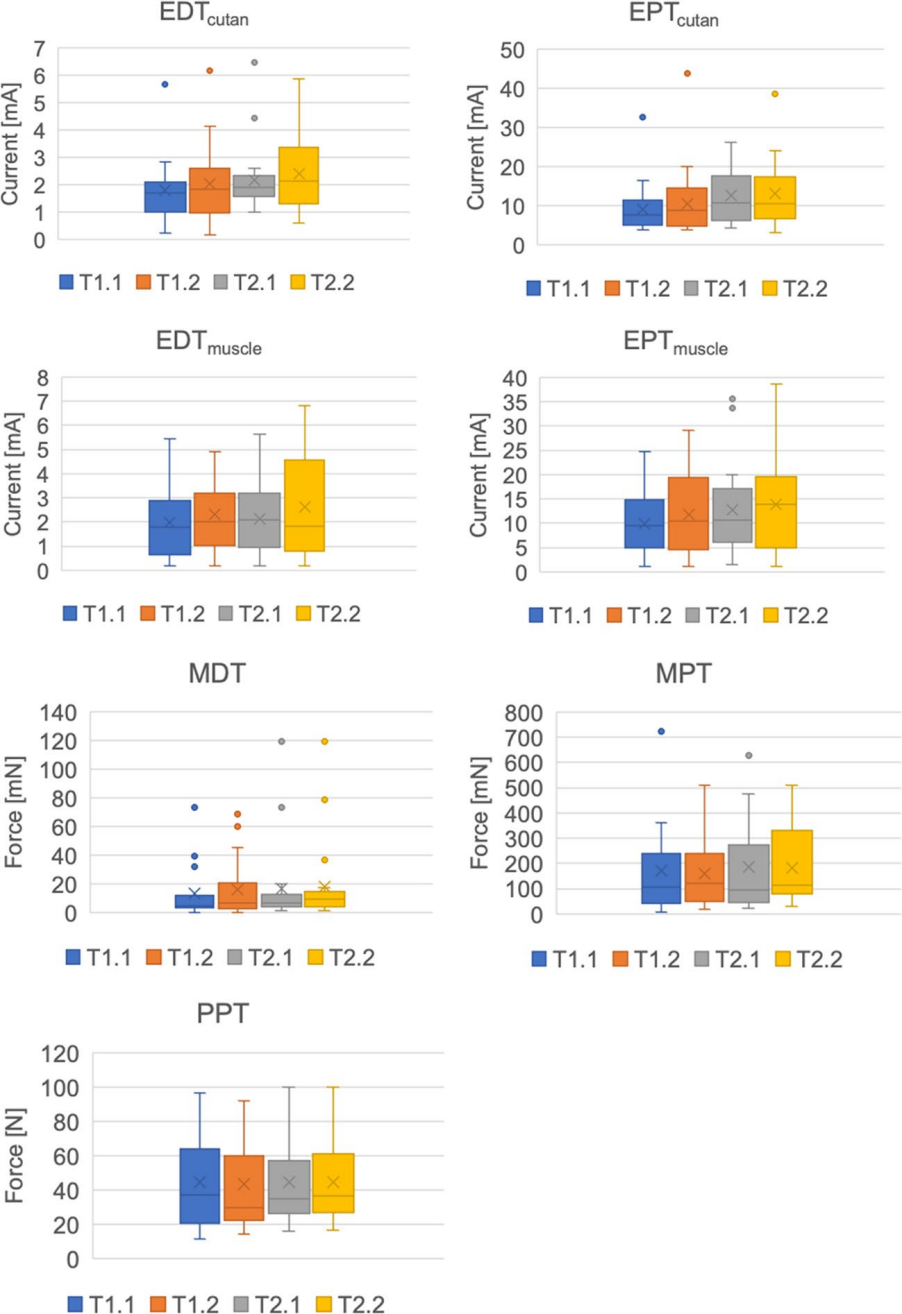
Box plots of EDT/EPT_cutan_, EDT/EPT_muscle_, and the QST parameters for T1.1–T2.2. Box plots with whiskers for EDT/EPT_cutan_, EDT/EPT_muscle_, and the QST parameters for T1.1–T2.2. The top end of the box corresponds to the first quartile, the bottom end to the third quartile, and the horizontal line in the box to the median. The arithmetic mean is represented by a cross. Outliers, more than 1.5 × interquartile range (IQR) above the first quartile or below the third quartile, indicated by the whiskers, are shown as dots

The detection and pain thresholds for the different modalities are presented in Table 2 and Fig. 3.

**Table 2:** Mean and standard deviation of all modalities for T1.1–T2.2

Parameters	T1.1	T1.2	T2.1	T2.2
	Mean ± SD	Mean ± SD	Mean ± SD	Mean ± SD
EDT_cutan_ [mA]	1.78 ± 1.17	2.02 ± 1.43	2.18 ± 1.25	2.39 ± 1.38
EPT_cutan_ [mA]	9.14 ± 6.48	10.56 ± 9.35	12.70 ± 7.25	13.05 ± 8.50
EDT_muscle_ [mA]	1.97 ± 1.46	2.31 ± 1.39	2.12 ± 1.48	2.62 ± 2.10
EPT_muscle_ [mA]	9.93 ± 6.30	11.75 ± 8.22	12.69 ± 9.26	13.93 ± 10.22
MDT [mN]	13.52 ± 18.57	15.69 ± 20.13	16.51 ± 28.76	18.32 ± 29.45
MPT [mN]	170.07 ± 174.15	160.40 ± 132.41	188.01 ± 177.76	182.92 ± 150.93
PPT [N]	44.45 ± 26.77	43.55 ± 26.39	44.40 ± 22.36	44.35 ± 22.18

*SD*, standard deviation

The five reported pain qualities most often mentioned by the participants, in descending order, are shown in Table 3

**Table 3:** Reported pain quality for EPT_cutan_, EPT_muscle_, MPT, and PPT for T1.1–T2.2

Reported pain quality	EPT_cutan_	EPT_muscle_	MPT	PPT
1st	Stinging (40%)	Stinging (14%)	Stinging (41%)	Pulling (25%)
2nd	Pricking (19%)	Pulling (14%)	Pricking (29%)	Pressure (18%)
3rd	Burning (12%)	Cramping (8%)	Sharp (13%)	Pushing (14%)
4th	Pinching (5%)	Radiating (6%)	Pushing (5%)	Burning (13%)
5th	Pulling (5%)	Twitching (6%)	Vibrating (5%)	Stinging (9%)
	Other (16%)	Other (52%)	Other (8%)	Other (23%)

### Distribution of EDT/EPT_cutan_*, *EDT/EPT_muscle_*, *and the QST parameters

The distribution properties of EDT/EPT_cutan_, EDT/EPT_muscle_, and the QST parameters were evaluated. Based on skewness, kurtosis, and Kolmogorov–Smirnov d statistics for raw and log-transformed data, the data that better approximated the normal distribution were chosen [36]. Based on this approach, the log transformation was applied for EDT/EPT_cutan_, MDT, MPT, and PPT, while raw data were utilized for EDT/EPT_muscle_ for the statistical analysis (Online Resource Table S1).

### Absolute differences for intra- and inter-session comparison

No significant mean difference (*p* ≤ 0.05) was observed for intra-session (T1.1–T1.2) EDT/EPT_cutan_, EDT/EPT_muscle_, and the QST parameters (Table 4). The inter-session (T1.1–T2.1) mean difference of EPT_cutan_ was significantly different from zero. Differences for all other modalities were non-significant (Table 5).

**Table 4:** Intra-session reliability of EDT/EPT_cutan_, EDT/EPT_muscle_, and QST parameters. T1.1 first measurement on day 1; T1.2 s measurement on day 1; level of significance: *p ≤ 0.001. *CI*, confidence interval; *EDT*, electrical detection threshold; *EPT*, electrical pain threshold (muscle, intramuscular; cutan, epidermis/dermis); *ICC*, intraclass correlation coefficient; *LoAs*, limit of agreements; *MDT*, mechanical detection threshold; *MPT*, mechanical pain threshold; *PPT*, pressure pain threshold; raw, based on raw data; *SD*, standard deviation; *SEM*, standard error of measurement

Parameters	Difference (T1.1–T1.2)		*p*	ICC	*p*		LoAs	SEM
	Mean ± SD	(95% CI)		ICC		(95% CI)	Lower–upper LoA	
Log EDT_cutan_ [mA]	− 0.03 ± 0.17	(− 0.11 to 0.05)	0.472	0.88	< 0.001*	(0.72 to 0.95)	− 2.19 to 1.73 (raw)	0.23 (raw)
Log EPT_cutan_ [mA]	− 0.04 ± 0.14	(− 0.11 to 0.03)	0.295	0.85	< 0.001*	(0.65 to 0.94)	− 8.38 to 5.37 (raw)	0.80 (raw)
EDT_muscle_ [mA]	− 0.27 ± 0.97	(− 0.73 to 0.20)	0.246	0.76	< 0.001*	(0.49 to 0.90)	− 2.17 to 1.64	0.22
EPT_muscle_ [mA]	− 1.35 ± 4.57	(− 3.55 to 0.85)	0.215	0.80	< 0.001*	(0.55 to 0.92)	− 10.31 to 7.61	1.05
Log MDT [mN]	− 0.05 ± 0.28	(− 0.18 to 0.08)	0.431	0.89	< 0.001*	(0.76 to 0.96)	− 23.38 to 19.05 (raw)	2.42 (raw)
Log MPT [mN]	− 0.04 ± 0.29	(− 0.17 to 0.10)	0.568	0.79	< 0.001*	(0.56 to 0.91)	− 139.85 to 159.18 (raw)	17.06 (raw)
Log PPT [N]	0.00 ± 0.10	(− 0.04 to 0.05)	0.855	0.93	< 0.001*	(0.84 to 0.97)	− 17.24 to 19.04 (raw)	2.07 (raw)

**Table 5:** Inter-session reliability of EDT/EPT_cutan_, EDT/EPT_muscle_, and QST parameters. T1.1 first measurement day 1; T2.1 first measurement day 2; level of significance: *p ≤ 0.001, **p ≤ 0.05. *CI*, confidence interval; *EDT*, electrical detection threshold; *EPT*, electrical pain threshold (muscle, intramuscular; cutan, epidermis/dermis); *ICC*, intraclass correlation coefficient; *LoAs*, limit of agreements; *MDT*, mechanical detection threshold; *MPT*, mechanical pain threshold; *PPT*, pressure pain threshold; raw, based on raw data; *SD*, standard deviation; *SEM*, standard error of measurement

Parameters	Difference (T1.1–T2.1)		*p*	ICC	*p*	(95% CI)	LoAs	SEM
	Mean ± SD	(95% CI)		ICC			Lower–upper LoA	
Log EDT_cutan_ [mA]	− 0.13 ± 0.35	(− 0.29 to 0.03)	0.110	0.08	0.35	(− 0.31 to 0.48)	− 3.65 to 2.87 (raw)	0.37 (raw)
Log EPT_cutan_ [mA]	− 0.14 ± 0.26	(− 0.27 to − 0.02)	0.024**	0.36	0.03	(− 0.04 to 0.67)	− 19.76 to 12.63 (raw)	1.85 (raw)
EDT_muscle_ [mA]	− 0.15 ± 2.02	(− 1.09 to 0.80)	0.746	0.08	0.37	(− 0.37 to 0.49)	− 4.11 to 3.81	0.45
EPT_muscle_ [mA]	− 2.76 ± 9.60	(− 7.25 to 1.74)	0.215	0.26	0.12	(− 0.18 to 0.62)	− 21.57 to 16.06	2.15
Log MDT [mN]	− 0.11 ± 0.39	(− 0.29 to 0.07)	0.212	0.74	< 0.001*	(0.46 to 0.89)	− 40.93 to 34.96 (raw)	4.33 (raw)
Log MPT [mN]	− 0.07 ± 0.33	(− 0.22 to 0.09)	0.376	0.75	< 0.001*	(0.47 to 0.89)	− 249.90 to 214.01 (raw)	26.46 (raw)
Log PPT [N]	− 0.03 ± 0.18	(− 0.11 to 0.05)	0.465	0.75	< 0.001*	(0.48 to 0.89)	− 32.88 to 32.98 (raw)	3.76 (raw)

### Intraclass correlation coefficients

Excellent intra-session ICCs were observed for EDT/EPT_cutan_, EDT/EPT_muscle_, and the QST parameters (ranging from ICC 0.76 to ICC 0.93; *p* ≤ 0.001) (Tables 4 and 5).

The inter-session ICCs were good for MDT, MPT, and PPT (ranging from ICC 0.74 to ICC 0.75; *p* ≤0.001) and poor for EDT/EPT_cutan_ and EDT/EPT_muscle_ (ranging from ICC 0.08 to ICC 0.36). The lowest values were observed for EDT_cutan_ and EDT_muscle_ (ICC 0.08; *p* = 0.35 and ICC 0.08; *p* = 0.37) (Tables 4 and 5).

### Bland–Altman plots

**Fig. 4:**
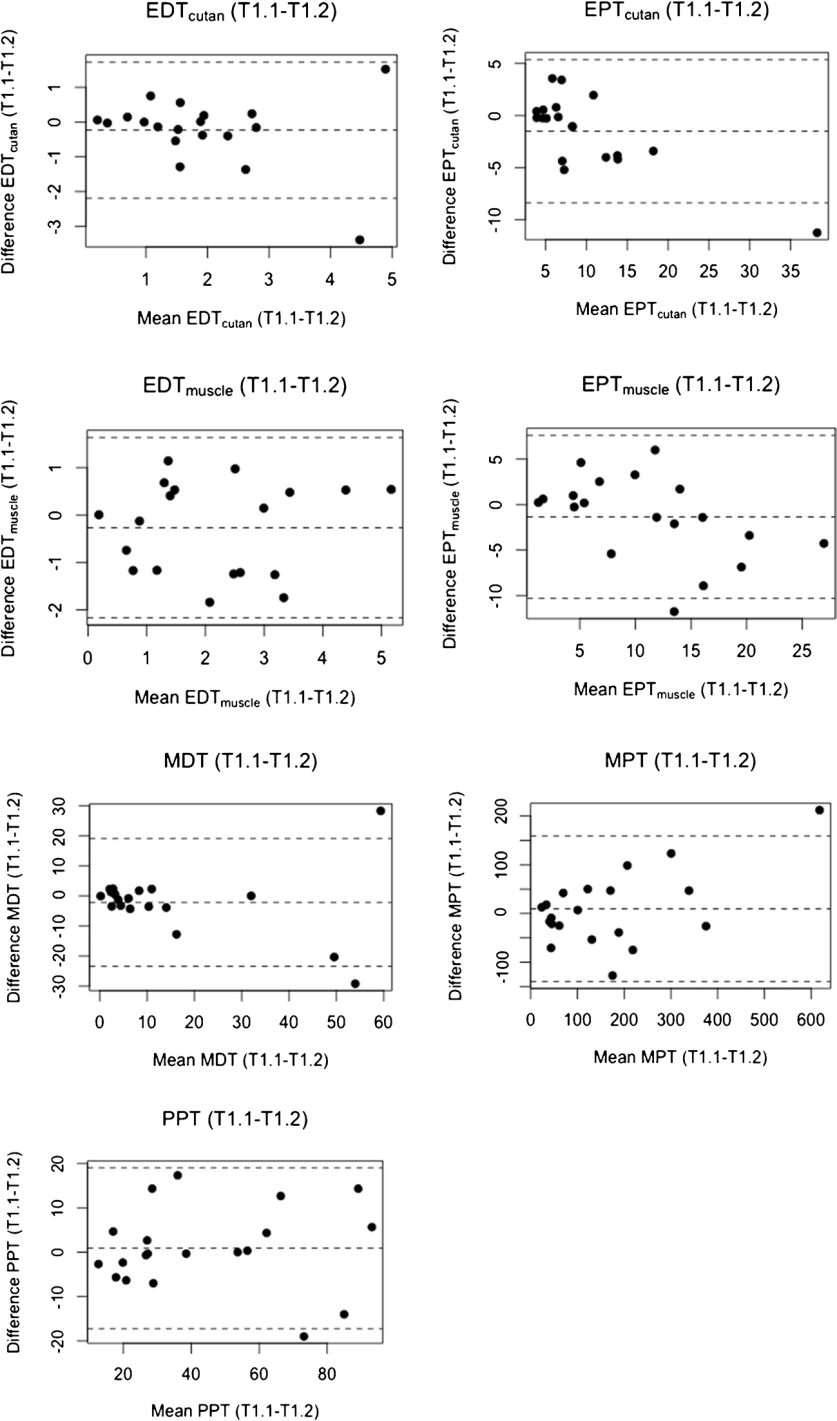
Bland–Altman plots of EDT/EPT_cutan_, EDT/EPT_muscle_, and the QST parameters for intra-session (T1.1–T1.2)*.* Bland–Altman plots for EDT/EPT_cutan_, EDT/EPT_muscle_, and the QST parameters with differences between T1.1 and T1.2 values (vertical axis) plotted against the mean of the T1.1 and T1.2 values (horizontal axis) of each participant. The middle-dashed line corresponds to the mean difference between T1.1 and T1.2 of all participants. The upper and lower dashed lines represent the limit of agreements (LoAs) (mean difference ± 1.96 × SD)

**Fig. 5:**
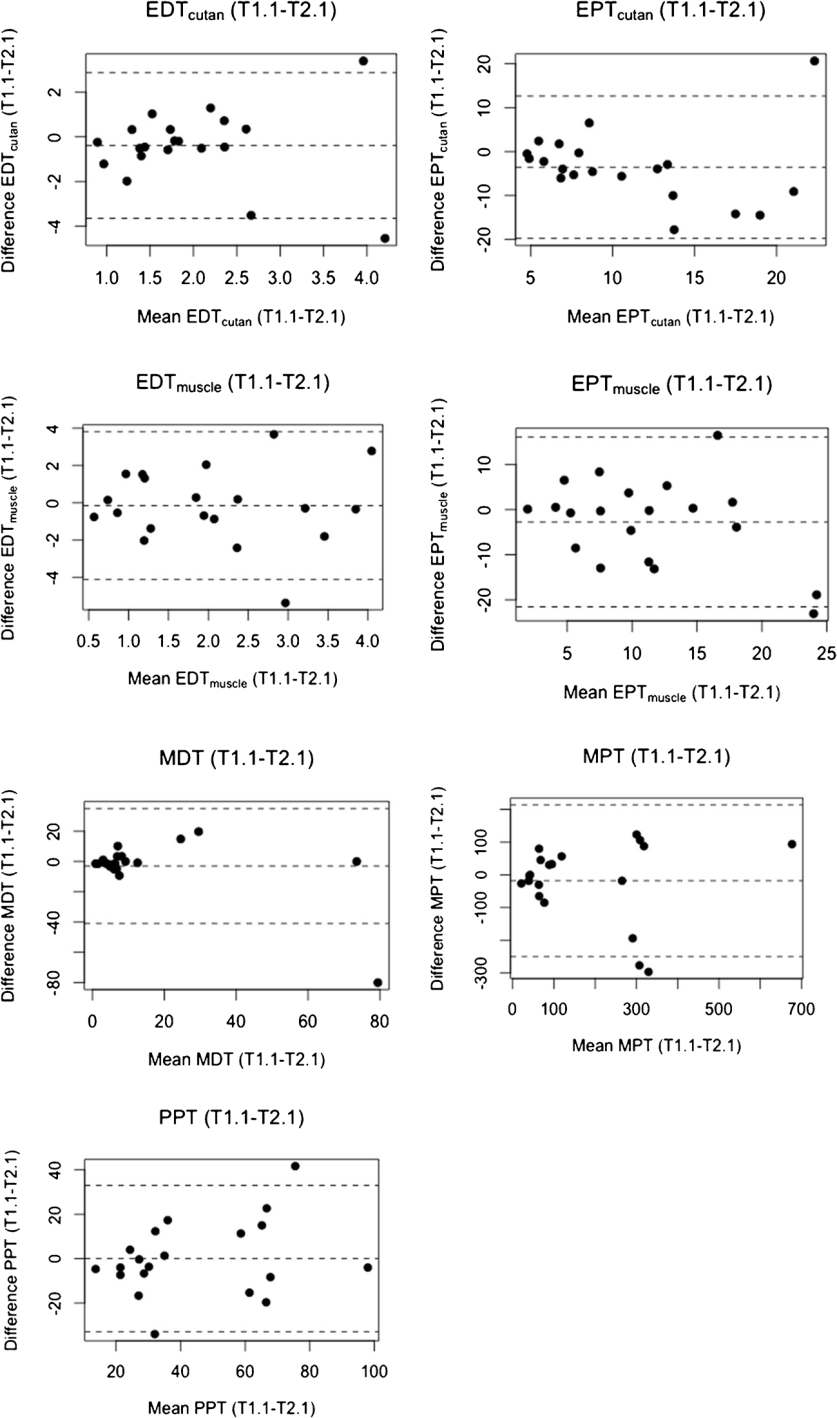
Bland–Altman plots of the inter-session EDT/EPT_cutan_, EDT/EPT_muscle_, and the QST parameters (T1.1–T2.1). Bland–Altman plots for EDT/EPT_cutan_, EDT/EPT_muscle,_ and the QST parameters with differences between T1.1 and T2.1 value (vertical axis) plotted against the mean of the T1.1 and T2.1 values (horizontal axis) of each participant. The middle-dashed line corresponds to the mean difference between T1.1 and T2.1 of all participants. The upper and lower dashed lines represent the limit of agreements (LoAs) (mean difference ± 1.96 × SD)

The Bland–Altman LoA plots for the different modalities for intra- and inter-session are presented in Figs. 4 and 5 for the raw data.

The intra-session LoAs for the different modalities varied relatively substantially with the lowest values for EDT_cutan_ and EDT_muscle_ and the highest for MPT. No systematic or proportion bias was evident for any modality.

The inter-session LoAs for the different modalities varied more than for intra-session. Again, the lowest values were observed for EDT_cutan_ and EDT_muscle_ and the highest for MPT. No proportion bias is evident, but a systematic bias is present for EPT_cutan_ with higher values for EPT_cutan_ 2.1.

### Standard error of measurement

The intra-session SEM were lower compared to the inter-session, indicating that the intra-session measurements are more precise (Tables 4 and 5).

### Correlation between modalities

No correlation was found between mechanical and electrical detection thresholds (*r* =  − 0.09; *p* = 0.646). In contrast, medium to strong correlations were observed between mechanical and electrical pain thresholds with *r* = 0.80 and *p* < 0.001 for EPT_cutan_ and MPT and *r* = 0.44 and *p* = 0.027 for EPT_muscle_ and PPT.

## Discussion

This study aimed to test the intra- and inter-session reliability of EDT and EPT of cutaneous and muscle primary afferents originating in the lower back. The reliability parameters were compared to those of well-established QST parameters such as MDT, MPT, and PPT. The current study is, to our knowledge, the first investigating the reliability of electrical stimuli of cutaneous and muscle primary afferents in the lower back.

### Detection and pain thresholds of cutaneous and muscle electrical stimulation and QST parameters

In the present study, the current required for EDT and EPT in the epidermis/dermis above the erector spinae muscle was similar to values obtained in the subcutis above the rectus abdominis muscle with two unipolar needle electrodes and a distance of 5 mm between the needle tips [1]. Further, the EDT in the rectus abdominis muscle was similar to the present findings in the erector spinae muscle, while the intramuscular EPT was significantly higher in the previous study. In a study by Schilder and colleagues, [39] the EPT, assessed using a bipolar concentric needle, was similar in the multifidus muscle to the present findings in the erector spinae muscle. In contrast, the EDT in the multifidus muscle was lower than the one in the erector spinae muscle in the current study. Methodological differences such as inter-electrode distance, leading to differences in spatial summation [28, 33], needle type (bipolar vs. unipolar), stimulus duration (2 ms in [39] vs. 0.04 ms in [1] and the current study), and the use of different muscles might explain divergent findings across studies.

The pain quality for EPT_cutan_ in the current study was most often “stinging” and “pricking,” most probably corresponding to the stimulation of Aδ fibers [7, 20], and with the 3rd most common sensation “burning” assumably corresponding to the stimulation of nociceptive C fibers [9, 10], in line with findings of Schilder and colleagues [40]. Pathophysiologically, “burning” pain quality is also considered as a prototypical descriptor for neuropathic pain [8].

For EPT_muscle_, the sensation seemed more ambiguous with stimulus qualities “stinging,” “pulling,” and “cramping” most often mentioned. These sensations were different to findings of using stimuli of an intensity of twice the magnitude of the individual pain threshold in the multifidus muscle, which elicited sensations of “deep pain” (“beating,” “throbbing,” and “pounding”) [40].

MDT in the present study are in line with the findings of a study in which testing sites were paraspinal and in the posterior axillary line between Th10 and L3 [31]. In contrast, MPT in the present study showed lower values. The different application sites and soft tissue thickness likely explain these differences. “Stinging,” “pricking,” and “sharp” were the most frequent pain qualities for MPT in the present study, whereas the cited study did not assess pain qualities but defined MPT by the perception of a “sharp” sensation. Further, the previous study observed similar PPT to the present study, with the application either over the lumbar latissimus dorsi or the gluteal muscle, while Balaguier and colleagues [5] measured higher PPT, with the application over 14 different anatomical sites in the lumbar region between L1-L5, 3-6 cm lateral to the spinous processes. The different application sites and respective soft tissue thickness, as well as the different pain threshold definition in the study by Balaguier and colleagues (“when the pressure becomes painful'”), might explain the difference. The most frequent pain qualities for PPT were pulling, pressing, and pushing in the current study. It seems that no previous study has investigated the quality of sensation at the PPT.

### Intra-session test–retest reliability

Excellent intra-session test–retest reliability (assessed by using ICC and *t*-test) was observed in the present study for all QST parameters, as well as cutaneous and intramuscular stimulations. Relatively good agreement in the Bland–Altman plots and relatively low SEM for intra-session comparison support the findings of the excellent ICCs.

For PPT, the intra-session reliability (assessed using ICC) in the meta-analysis of Nuwailati and colleagues [30] that included testing sites at the neck, shoulder, and extremities is in line with the current study. The high intra-session reliability of cutaneous and intramuscular electrical stimulations as found in the present study, which are comparable to the reliability of the very established QST parameter PPT, strengthens the validity of the electrical stimulations.

### Inter-session test–retest reliability

The inter-session reliability (assessed using ICC) was poor for EDT/EPT_cutan_ and EDT/EPT_muscle_, while EPT_cutan_ and EPT_muscle_ had higher ICCs than EDT_cutan_ and EDT_muscle_. The reliability for the well-established QST modalities MDT, MPT, and PPT was good and just below the threshold to excellent.

In a previous study by Aasvang and colleagues [1], the reliability of the EDT and EPT of the subcutis and rectus abdominis muscle in a 10-day test–retest study was determined. The observed poor reliability (assessed using ICCs and Bland–Altman plots) for EPT_cutan_ and EDT_muscle_ is in line with the present study. In contrast to the present study, Aasvang and colleagues observed good respectively, excellent ICCs for EDT_cutan_ and EPT_muscle_.

The lower reliability for EPT_muscle_ in the current study might be explained by the numerous layers of fasciae around the rectus abdominis muscle [43]. Because the innervation density in fasciae is higher than that in muscle [6], this might lead to less threshold variation with repeated needle placement. The higher reliability for EDT_cutan_ compared to the current study could be due to the stimulation of the subcutis instead of the dermis.

Nothnagel and colleagues [29] observed a lower inter-session reliability (for MDT and a slightly lower inter-session reliability for MPT over a 10-week period in the paraspinal lumbar area compared to the present study). This difference might be explained by the longer time interval between the sessions. This notion that the duration of the inter-session interval matters is supported by previous studies investigating inter-session reliability of PPT that, as the present study, observed good inter-session reliability (assessed using ICC) for PPT at the lower back over a period of 10 weeks [29] or 4 months [23] whereas a study assessing PPT on the lumbar erector spinae with a time interval between 2 and 7 days [24] observed excellent reliability according to ICC.

### Correlation between modalities

No relationship between electrical and mechanical detection thresholds was observed. A potential explanation for this is that mechanical stimuli activate mechanoreceptors (e.g., Merkel’s disks, Meissner’s corpuscles, Ruffini endings, and Pacinian corpuscles) that convey information through Aβ-fibers while electrical stimulation bypasses the receptors and directly activates nerves fibers that might be mechanosensitive or not [2]. In contrast, pain thresholds showed medium to strong correlations between mechanical and electrical stimulation, perhaps indicating that in this instance, the respective receptor modality is less important. Indeed, a large proportion of an individual’s pain sensitivity, at least as thresholds are concerned, is independent of modality [26].

### Strengths and limitations

Our study lays the groundwork by demonstrating the reliability of the method, and future studies can build upon this foundation to explore painful conditions and the relationship between electrical stimulation, allodynia, and hyperalgesia. Excellent intra-session ICCs and good agreement enable further investigations of cutaneous and muscle primary afferents including using paradigms that depend on repeated stimulations such as CPM investigations.

The poor inter-session reliability for cutaneous and intramuscular electrical stimulation is likely due to the need to newly position the needle electrodes in the tissue, despite the positioning being ultrasound-guided. Therefore, measurement comparison between different days is not recommended.

A further limitation is that electrical stimulation does not allow to selectively stimulate small-diameter fibers without stimulating large-diameter fibers. W.r.t. the skin, the epidermis contains mostly small-diameter fibers, which is exploited for selective stimulation of nociceptors using concentric intra-epidermal electrodes [18]. We mimicked this in the present study by inserting the cutaneous needles into the epidermis/dermis. However, this is not applicable for intra-muscular electrical stimulation. To keep cutaneous and intra-muscular electrical stimulation as similar as possible, we used two unipolar needles rather than a concentric electrode. Given the observation that also for the cutaneous stimulation, the currents for the EDT were much lower than that for the EPT, and large-diameter low-threshold mechanoreceptors were likely stimulated.

## Conclusions

With this study, excellent intra-session ICCs and relatively good agreement for cutaneous and intramuscular EDT and EPT were demonstrated. These ICCs are as good as for well-established QST paradigms, which were confirmed here for the lower back. In contrast, poor inter-session ICCs were observed for cutaneous and intramuscular EDT and EPT, probably due to needle repositioning. These findings allow cutaneous and intramuscular EDT/EPT to be used as static or dynamic QST measures for further investigations, e.g., of central sensitization at the spinal or supraspinal level. Further research is needed to investigate the utility of electrical stimulation in examining painful conditions. Specifically, future investigations should address the relationship between changes in electrical and mechanical stimulation data on z-score sensory profiles (e.g., gain of function, loss of function) in populations with chronic pain in order to better understand the mechanisms of mechanical allodynia and hyperalgesia.

## Supplementary Information

Below is the link to the electronic supplementary material.

Supplementary file1 (PDF 51 KB)

Supplementary file2 (PDF 21 KB)

## Data Availability

The datasets that support the findings of this study are available from the corresponding author upon reasonable request.
