# Supplementary figures and images for: Intra- and inter-session reliability of electrical detection and pain thresholds of cutaneous and muscle primary afferents in the lower back of healthy individuals
Source: Pflugers Arch. 2023 Aug 25;475(10):1211–23. doi: 10.1007/s00424-023-02851-7 (PMC10499933; doi:10.1007/s00424-023-02851-7)

**Fig. S1**

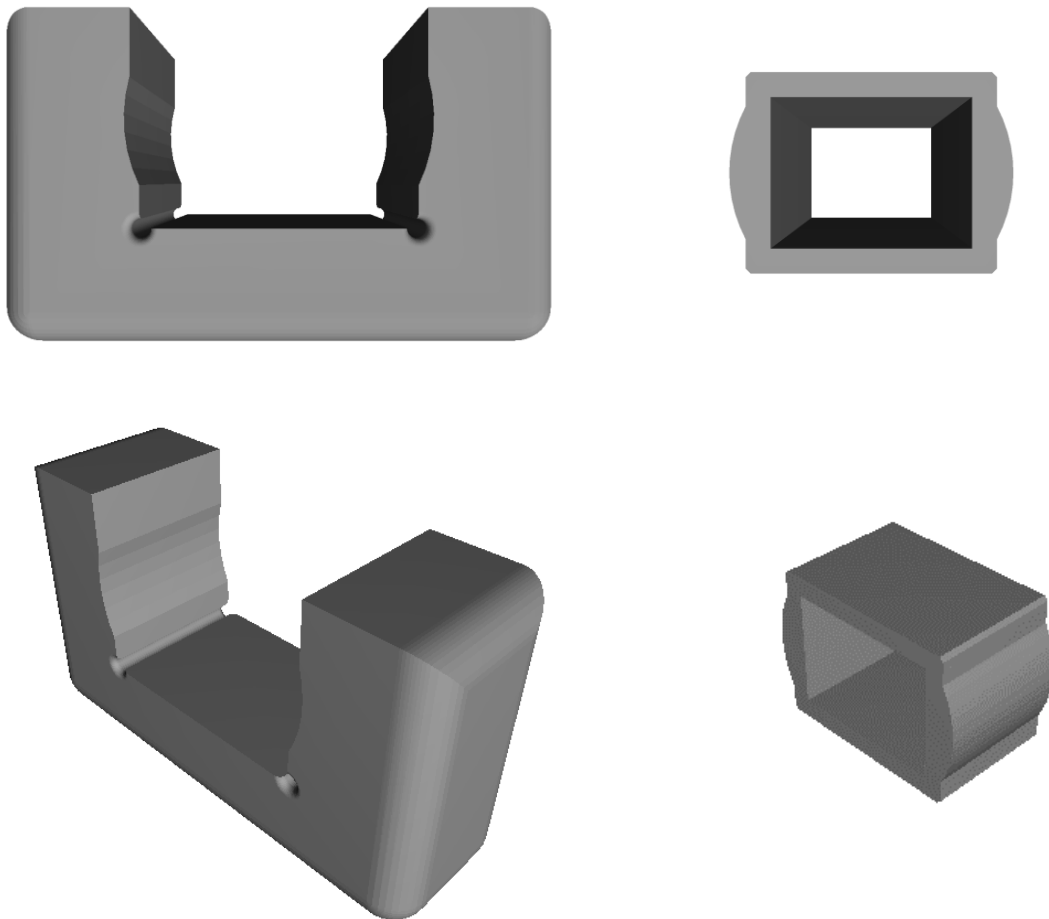

Custom-made needle-guiding device

Supplement: Supplementary file 1 — Supplementary file1 (PDF 51 KB) [file 424_2023_2851_MOESM1_ESM.pdf]
