# Supplementary material for: Intra- and inter-session reliability of electrical detection and pain thresholds of cutaneous and muscle primary afferents in the lower back of healthy individuals
Source: Pflugers Arch. 2023 Aug 25;475(10):1211–23. doi: 10.1007/s00424-023-02851-7 (PMC10499933; doi:10.1007/s00424-023-02851-7)
Supplement: Supplementary file 2 — Supplementary file2 (PDF 21 KB) [file 424_2023_2851_MOESM2_ESM.pdf]

Table S1. Properties of the threshold distributions for measurement T1.1

Modality	Data	Skewness	Kurtosis	K-S d	Weighted Ratio (Raw/Log)	Recommended data transformation
EDT_{cutan} [mA]	Raw	1.73	7.05	0.68	3.8	Log
	Log	-0.81	3.92	0.36		
EPT_{cutan} [mA]	Raw	2.52	9.75	1.00	4.0	Log
	Log	0.84	3.55	0.72		
EDT_{muscle} [mA]	Raw	0.79	3.02	0.60	2.5	Raw
	Log	-0.77	2.61	0.26		
EPT_{muscle} [mA]	Raw	0.48	2.68	0.88	1.0	Raw
	Log	-0.91	2.96	0.59		
MDT [mN]	Raw	2.05	6.57	0.90	4.5	Log
	Log	-0.44	3.65	0.57		
MPT [mN]	Raw	1.76	6.08	1.00	3.9	Log
	Log	-0.34	2.67	0.88		
PPT [N]	Raw	0.61	2.18	1.00	3.0	Log
	Log	-0.11	1.86	0.85		

EDT = electrical detection threshold, EPT = electrical pain threshold (muscle = intramuscular, cutan = epidermis/dermis), K-S = Kolmogorov-Smirnov, MDT = mechanical detection threshold, MPT = mechanical pain threshold, PPT = pressure pain threshold
